# Antimicrobial Resistance of *Shigella flexneri* Serotype 1b Isolates in China

**DOI:** 10.1371/journal.pone.0129009

**Published:** 2015-06-03

**Authors:** Xianyan Cui, Chaojie Yang, Jian Wang, Beibei Liang, Shengjie Yi, Hao Li, Hongbo Liu, Peng Li, Zhihao Wu, Jing Xie, Leili Jia, Rongzhang Hao, Ligui Wang, Yuejin Hua, Shaofu Qiu, Hongbin Song

**Affiliations:** 1 Department of Nuclear-Agricultural Sciences, Zhejiang University, Hangzhou, China; 2 Department of Disease Control and Prevention, Academy of Military Medical Sciences, Beijing, China; Ross University School of Veterinary Medicine, SAINT KITTS AND NEVIS

## Abstract

*Shigella flexneri* serotype 1b is among the most prominent serotypes in developing countries, followed by serotype 2a. However, only limited data is available on the global phenotypic and genotypic characteristics of *S*. *flexneri* 1b. In the present study, 40 *S*. *flexneri* 1b isolates from different regions of China were confirmed by serotyping and biochemical characterization. Antimicrobial susceptibility testing showed that 85% of these isolates were multidrug-resistant strains and antibiotic susceptibility profiles varied between geographical locations. Strains from Yunnan were far more resistant than those from Xinjiang, while only one strain from Shanghai was resistant to ceftazidime and aztreonam. Fifteen cephalosporin resistant isolates were identified in this study. ESBL genes (*bla*_SHV_, *bla*_TEM_, *bla*_OXA_, and *bla*_CTX-M_) and ampC genes (*bla*_MOX,_ *bla*_FOX,_ *bla*_MIR(ACT-1),_ *bla*_DHA,_ *bla*_CIT_ and *bla*_ACC_) were subsequently detected among the 15 isolates. The results showed that these strains were positive only for *bla*_TEM_, *bla*_OXA_, *bla*_CTX-M_, *intI1*, and *intI2*. Furthermore, pulsed-field gel electrophoresis (PFGE) analysis showed that the 40 isolates formed different profiles, and the PFGE patterns of Xinjiang isolates were distinct from Yunnan and Shanghai isolates by one obvious, large, missing band. In summary, similarities in resistance patterns were observed in strains with the same PFGE pattern. Overall, the results supported the need for more prudent selection and use of antibiotics in China. We suggest that antibiotic susceptibility testing should be performed at the start of an outbreak, and antibiotic use should be restricted to severe *Shigella* cases, based on resistance pattern variations observed in different regions. The data obtained in the current study might help to develop a strategy for the treatment of infections caused by *S*. *flexneri* 1b in China.

## Introduction

*Shigella*, the causative agent of human shigellosis, is a significant public health burden. Worldwide, 164.7 million *Shigella* episodes per year are estimated, of which 163.2 million are in developing countries. The majority of the infections and deaths caused by *Shigella* are in children <5 years old [1]. Moreover, a surveillance study in six Asian countries showed the global disease burden caused by shigellosis might be much higher than these estimates [2]. In China, shigellosis is one of the top four notifiable infectious diseases, with nearly half a million cases annually [3].

*Shigella* species are in fact clones of *Escherichia coli* [4]. Based on biochemical and serological properties, the genus *Shigella* is divided into four species or subgroups: *S*. *dysenteriae*, *S*. *flexneri*, *S*. *boydii*, and *S*. *sonnei*, among which, *S*. *flexneri* is the predominant species in developing countries [1]. A previous study showed that the annual shigellosis morbidity rate was 20.3 cases per 100,000 people in China using the national surveillance data from 2009, and *S*. *flexneri* (67.3%) and *S*. *sonnei* (32.7%) were two major causative species [5]. Because *Shigella* strains are all negative for H antigen, *Shigella* serotyping is based on the O antigen only. To date, 20 *S*. *flexneri* serotypes (1a, 1b, 1c (or 7a), 1d, 2a, 2b, 2v, 3a, 3b, 4a, 4av, 4b, 5a, 5b, X, Xv, Y, Yv, 6, and 7b) have been recognized [6,7]. Some *S*. *flexneri* serotypes are more prevalent than others, and *S*. *flexneri* 1b is among the most commonly encountered serotypes in developing countries, followed by serotype 2a [1].

Pickering LK showed that antimicrobial susceptibility patterns of *Shigella* species, were influenced by geographic location and other factors (year, classes of antimicrobial agents, pressure exerted by antimicrobial use, source of isolates) in endemic regions [8]. However, comparisons of associations between geographical diversity and antimicrobial resistance of serotype 1b strains are largely lacking. The objective of the present study was to evaluate the antimicrobial resistance profiles of *S*. *flexneri* 1b isolates from different regions of China, and guide the selection of the most effective antimicrobial agents for shigellosis treatment.

## Materials and Methods

### Bacterial isolates, serotyping, and biochemical characterization

During our routine surveillance of bacillary dysentery, fecal samples from individual outpatients with diarrhea or dysentery were collected and screened for *Shigella* species in sentinel hospitals based on a national pathogen monitoring system. Samples were streaked directly onto *Salmonella-Shigella* (SS) agar and incubated overnight at 37°C. Resultant colonies were picked and streaked directly onto SS agar and again incubated overnight at 37°C. The colonies were subcultured on Luria–Bertani agar plates and incubated overnight at 37°C, and were then sent to our laboratory for further confirmation. This study was approved by the ethics committee of the Academy of Military Medical Sciences (China). Written informed consent was obtained from the patients involved in this study. The isolates were confirmed using API 20E test strips (bioMerieux Vitek, Marcy-l’Etoile, France) following the manufacturer’s recommendations. Serotype identification was performed using two serotyping kits: a kit including antisera specific for all type and group-factor antigens (Denka Seiken, Tokyo, Japan), and a monoclonal antibody reagent kit (Reagensia AB, Stockholm, Sweden), specific for all *S*. *flexneri* type and group-factor antigens. Serological reactions were performed using the slide agglutination test, as described previously [9].

### Antimicrobial susceptibility testing

MICs of 21 antimicrobial agents including ceftazidime (CAZ), ceftriaxone (CRO), cefepime (FEP), cefoperazone (CFP), cefazolin (CFZ), cefoxitin (FOX), imipenem (IPM), nitrofurantoin (NIT), piperacillin (PIP), ampicillin (AMP), ticarcillin (TIC), tetracycline (TE), tobramycin (TO), gentamicin (GEN), amikacin (AK), aztreonam (ATM), chloramphenicol (C), ticarcillin/clavulanic acid (TIM), levofloxacin (LEV), norfloxacin (NOR), and trimethoprim/sulfamethoxazole (SXT) were tested for each of the isolates. MICs were determined by broth microdilution using a 96-well microtiter plate (Sensititre, Thermo Fisher Scientific Ine, West Sussex, United Kingdom) according to the recommendations of the Clinical and Laboratory Standards Institute. *E*. *coli* ATCC 25922 was used as the control strain for susceptibility studies.

### Pulsed-field gel electrophoresis (PFGE)

The isolates were analyzed by PFGE following digestion with *Not*I to generate DNA fingerprinting profiles according to the procedures developed by the CDC PulseNet program [10]. *Salmonella enterica* serotype Braenderup H9812 was used as the molecular size marker. PFGE patterns were interpreted using BioNumerics (Version 6.0) software (Applied Maths, Sint-Martens-Latem, Belgium). A tree indicating relative genetic similarity was constructed based on the unweighted pair group method of averages (UPGMA) and a position tolerance of 1.2%.

### PCR amplification of the antibiotic-resistance determinants and integrons

PCR assays were carried out as described previously [11–16] to detect ESBL genes (*bla*_SHV_, *bla*_TEM_, *bla*_OXA_, and *bla*_CTX-M_) and ampC genes (*bla*_MOX_, *bla*_FOX_, *bla*_MIR(ACT-1)_, *bla*_DHA_, *bla*_CIT_ and *bla*_ACC_) which are responsible for resistance to cephalosporins. We also amplified the variable regions of class 1 and 2 integrons using primers listed in Table 1. The reverse primers aadA1, aadA2, aadA5, and cmlA1 were used with forward primer hep58 to amplify the gene cassettes of class 1 integron-positive strains. Forward primer hep74 and reverse primer hep51 were used to amplify the gene cassettes of class 2 integron-positive strains. Resultant PCR products were fully sequenced and analyzed by comparison with sequences in GenBank.

**Table 1:** Primers for detection of antibiotic-resistance determinants and integrons.

Target	Primer sequence (5′ to 3′)	Reference
**β-lactamases (bp)**		
*bla*_CTX-M-1 group_ (873 bp)	F: GGTTAAAAAATCACTGCGTC	Matar *et al*. [15]
	R: TTACAAACCGTCGGTGACGA	Matar *et al*. [15]
*bla*_CTX-M-9 group_ (868 bp)	F: AGAGTGCAACGGATGATG	Matar *et al*. [15]
	R: CCAGTTACAGCCCTTCGG	Matar *et al*. [15]
*bla*_CTX-M-2/8/25 group_ (221 bp)	F: ACCGAGCCSACGCTCAA	This study
	R: CCGCTGCCGGTTTTATC	This study
*bla*_TEM_ (1080 bp)	F: ATGAGTATTCAACATTTCCG	Tariq *et al*. [11]
	R: CCAATGCTTAATCAGTGAGG	Tariq *et al*. [11]
*bla*_OXA_ (890 bp)	F: ATTAAGCCCTTTACCAAACCA	Ahmed *et al*. [12]
	R: AAGGGTTGGGCGATTTTGCCA	Ahmed *et al*. [12]
*bla*_MOX_ (520 bp)	F: GCTGCTCAAGGAGCACAGGAT	F. Javier *et al*. [16]
	R: CACATTGACATAGGTGTGGTGC	F. Javier *et al*. [16]
*bla*_FOX_ (190 bp)	F: AACATGGGGTATCAGGGAGATG	F. Javier *et al*. [16]
	R: CAAAGCGCGTAACCGGATTGG	F. Javier *et al*. [16]
*bla*_MIR(ACT-1)_ (302 bp)	F: TCGGTAAAGCCGATGTTGCGG	F. Javier *et al*. [16]
	R: CTTCCACTGCGGCTGCCAGTT	F. Javier *et al*. [16]
*bla*_DHA_ (405 bp)	F: AACTTTCACAGGTGTGCTGGGT	F. Javier *et al*. [16]
	R: CCGTACGCATACTGGCTTTGC	F. Javier *et al*. [16]
*bla*_CIT_ (462 bp)	F: TGGCCAGAACTGACAGGCAAA	F. Javier *et al*. [16]
	R: TTTCTCCTGAACGTGGCTGGC	F. Javier *et al*. [16]
*bla*_ACC_ (346 bp)	F: AACAGCCTCAGCAGCCGGTTA	F. Javier *et al*. [16]
	R: TTCGCCGCAATCATCCCTAGC	F. Javier *et al*. [16]
**Integrons**		
*IntI1* (569 bp)	F: ACATGTGATGGCGACGCACGA	Pan *et al*. [14]
	R: ATTTCTGTCCTGGCTGGCGA	Pan *et al*. [14]
Class 1 integron variable region	hep58: TCATGGCTTGTTATGACTGT	This study
	hep59: GTAGGGCTTATTATGCACGC	This study
Class 1 integron variable region	hep58: TCATGGCTTGTTATGACTGT	This study
	aadA1: TGTCAGCAAGATAGCCAGAT	This study
Class 1 integron variable region	hep58: TCATGGCTTGTTATGACTGT	This study
	aadA2: TGATCTCGCCTTTCACAAA	This study
Class 1 integron variable region	hep58: TCATGGCTTGTTATGACTGT	This study
	aadA5: CATCTAACGCATAGTTGAGC	This study
Class 1 integron variable region	hep58: TCATGGCTTGTTATGACTGT	This study
	cmlA1: CAACGATTGGGATTTGACGTACTTT	This study
*IntI2* (789 bp)	F: CACGGATATGCGACAAAAAGGT	This study
	R: GTAGCAAACGAGTGACGAAATG	This study
Class 2 integron variable region	hep74: CGGGATCCCGGACGGCATGCACGATTTGTA	This study
	hep51: GATGCCATCGCAAGTACGAG	This study

## Results

### Bacterial isolation and biochemical characterization

We isolated and identified 40 *S*. *flexneri* 1b isolates from patients with either diarrhea or dysentery over the period from 2005 to 2013. The strain information is detailed in Table 2. Among them, 28 isolates were collected from Xinjiang from 2005–2012, 11 from Yunnan in 2009, and one from Shanghai in 2013. All *S*. *flexneri* serotype 1b strains examined possessed typical *S*. *flexneri* biochemical characteristics, and analysis of biochemical reactions indicated the presence of four biotypes among these serotype 1b strains (Table 3).

**Table 2:** Strain information of *S*. *flexneri* serotype 1b isolates from diarrheal patients.

Original No	Species	Serotype	Source	Origin	Isolation time
SH13sh024	*S*. *flexneri*	1b	Human	Shanghai	2013
2005126	*S*. *flexneri*	1b	Human	Xinjiang	2005
2005173	*S*. *flexneri*	1b	Human	Xinjiang	2005
2006230	*S*. *flexneri*	1b	Human	Xinjiang	2006
2008026	*S*. *flexneri*	1b	Human	Xinjiang	2008
2008046	*S*. *flexneri*	1b	Human	Xinjiang	2008
2008076	*S*. *flexneri*	1b	Human	Xinjiang	2008
2009158	*S*. *flexneri*	1b	Human	Xinjiang	2009
2009378	*S*. *flexneri*	1b	Human	Xinjiang	2009
2010026	*S*. *flexneri*	1b	Human	Xinjiang	2010
2010144	*S*. *flexneri*	1b	Human	Xinjiang	2010
2010230	*S*. *flexneri*	1b	Human	Xinjiang	2010
2010236	*S*. *flexneri*	1b	Human	Xinjiang	2010
2010294	*S*. *flexneri*	1b	Human	Xinjiang	2010
2010297	*S*. *flexneri*	1b	Human	Xinjiang	2010
2010315	*S*. *flexneri*	1b	Human	Xinjiang	2010
2011032	*S*. *flexneri*	1b	Human	Xinjiang	2011
2011033	*S*. *flexneri*	1b	Human	Xinjiang	2011
2011052	*S*. *flexneri*	1b	Human	Xinjiang	2011
2011065	*S*. *flexneri*	1b	Human	Xinjiang	2011
20110163	*S*. *flexneri*	1b	Human	Xinjiang	2011
20110176	*S*. *flexneri*	1b	Human	Xinjiang	2011
20110187	*S*. *flexneri*	1b	Human	Xinjiang	2011
2012059	*S*. *flexneri*	1b	Human	Xinjiang	2012
2012061	*S*. *flexneri*	1b	Human	Xinjiang	2012
2012063	*S*. *flexneri*	1b	Human	Xinjiang	2012
2012073	*S*. *flexneri*	1b	Human	Xinjiang	2012
2012133	*S*. *flexneri*	1b	Human	Xinjiang	2012
2012147	*S*. *flexneri*	1b	Human	Xinjiang	2012
CDSF2	*S*. *flexneri*	1b	Human	Yunnan	2009
CDSF3	*S*. *flexneri*	1b	Human	Yunnan	2009
CDSF4	*S*. *flexneri*	1b	Human	Yunnan	2009
CDSF5	*S*. *flexneri*	1b	Human	Yunnan	2009
CDSF6	*S*. *flexneri*	1b	Human	Yunnan	2009
CDSF7	*S*. *flexneri*	1b	Human	Yunnan	2009
CDSF8	*S*. *flexneri*	1b	Human	Yunnan	2009
CDSF9	*S*. *flexneri*	1b	Human	Yunnan	2009
CDSF10	*S*. *flexneri*	1b	Human	Yunnan	2009
CDSF11	*S*. *flexneri*	1b	Human	Yunnan	2009
CDSF12	*S*. *flexneri*	1b	Human	Yunnan	2009

**Table 3:** Biochemical patterns of *S*. *flexneri* serotype 1b strains.

Biotype	Total No. % (n = 40)	Xinjiang No. % (n = 28)	Yunnan No. % (n = 11)	Shanghai No. % (n = 1)
B1	4 (10%)	3 (10.7%)	0	1 (100%)
B2	20 (50%)	20 (71%)	0	0
B3	2 (5%)	2 (7%)	0	0
B4	14 (35%)	3 (10.7%)	11 (100%)	0

Biotype B1: mannose-, glucose-, and melibiose-positive; biotype B2: mannose-, glucose-, arabinose-, and melibiose-positive; biotype B3: mannose-, glucose-, and sucrose-positive; biotype B4: mannose-, glucose-, and arabinose-positive.

### Antimicrobial susceptibility testing

The 40 *S*. *flexneri* 1b isolates were tested for susceptibility to 21 antimicrobials (Table 4). Resistance to TIC or AMP was the most common (36/40, 90%), followed by TE (35/40, 87.5%), C (33/40, 82.5%), SXT (20/40, 50%), PIP (16/40, 40%), TIM (12/40, 30%), and ATM (1/40, 2.5%). None of the isolates were resistant to NIT, IPM, FEP, FOX, AK, TO, or GEN. In addition, strains with intermediate resistance to TIM, ATM, and PIP were also observed.

**Table 4:** Comparison of susceptibility to 21 antibiotics among *S*. *flexneri* 1b isolates from different regions.

	Antimicrobial resistance rate No. (%)			
Antibiotic	Total (n = 40)	Xinjiang (n = 28)	Yunnan (n = 11)	Shanghai No. %(n = 1)
CAZ	1 (2.5%)	0	0	1 (100%)
CRO	14 (35%)	3 (10.7%)	11 (100%)	0
PIP	16 (40%)	4 (14%)	11 (100%)	1 (100%)
TE	36 (90%)	24 (85.7%)	11 (100%)	1 (100%)
CFP	14 (35%)	3 (10.7%)	11 (100%)	0
CFZ	14 (35%)	3 (10.7%)	11 (100%)	0
TIC	35 (87.5%)	24 (85.7%)	11 (100%)	0
TIM	12 (30%)	1 (3.6%)	11 (100%)	0
ATM	1 (2.5%)	0	0	1 (100%)
AMP	36 (90%)	24 (85.7%)	11 (100%)	1 (100%)
C	34 (85%)	22 (78.5%)	11 (100%)	0
SXT	20 (50%)	9(32.1%)	11 (100%)	0
LEV	0	0	0	0
FEP	0	0	0	0
IPM	0	0	0	0
NIT	0	0	0	0
FOX	0	0	0	0
TO	0	0	0	0
GEN	0	0	0	0
NOR	0	0	0	0
AK	0	0	0	0

Multidrug resistance (MDR; resistant to three or more classes of antimicrobials) was detected in 85% of the *S*. *flexneri* 1b isolates, and these isolates had diverse antibiotic-resistance profiles. About 78% of the isolates from Xinjiang were MDR isolates, dominated by resistance to AMP/TIC/TE/C (10/28, 35.7%), followed by AMP/TIC/TE/C/SXT (7/28, 25%), and CFP/CRO/CFZ/PIP/AMP/TIC/TE/C/TIM (3/28, 10.7%). All of the isolates from Yunnan presented the same resistance pattern, CFP/CRO/CFZ/PIP/AMP/TIC/TE/C/TIM/SXT, and the single strain from Shanghai exhibited a resistance profile of CAZ/CFP/CRO/CFZ/PIP/AMP/TIC/ATM (Table 5).

**Table 5:** Dominant antimicrobial resistance profiles of 40 *S*. *flexneri* 1b isolates from different regions of China.

Antimicrobial resistance profiles	Total	Xinjiang	Sichuan
	No. % (n = 40)	No. % (n = 28)	No. % (n = 11)
Sensitive (n = 2)	2 (5%)	2 (7%)	0
TE (n = 2)	2 (5%)	2 (7%)	0
AMP/TIC (n = 1)	1 (2.5%)	1 (3.6%)	0
AMP/TIC/C (n = 1)	1 (2.5%)	1 (3.6%)	0
AMP/TIC/TE/C (n = 10)	10 (25%)	10 (35.7%)	0
AMP/TIC/TE/C/SXT (n = 7)	7 (17.5%)	7 (25%)	0
PIP/AMP/TIC/TE/SXT (n = 1)	1 (2.5%)	1 (3.6%)	0
AMP/TIC/TE/C/TIM/SXT (n = 1)	1 (2.5%)	1 (3.6%)	0
CAZ/CFP/CRO/CFZ/PIP/AMP/TIC/ATM (n = 1)	1 (2.5%)	0	0
CFP/CRO/CFZ/PIP/AMP/TIC/TE/C/TIM (n = 3)	3 (7.5%)	3 (10.7%)	0
CFP/CRO/CFZ/PIP/AMP/TIC/TE/C/TIM/SXT (n = 11)	11 (27.5%)	0	11 (100%)

### Molecular analysis of antibiotic-resistance determinants and integrons

In this study, fifteen isolates were found to be resistant to cephalosporin antibiotics, and these strains were tested for antibiotic-resistance determinants and integrons, including the *bla*_SHV_, *bla*_TEM_, *bla*_OXA_, *bla*_CTX-M_, *bla*_MOX_, *bla*_FOX_, *bla*_MIR(ACT-1)_, *bla*_DHA_, *bla*_CIT_, *bla*_ACC_, *intI1*, and *intI2* gene regions (Table 6). PCR results showed that all 15 tested isolates were negative for *bla*_SHV_, *bla*_MOX_, *bla*_FOX_, *bla*_MIR(ACT-1)_, *bla*_DHA_, *bla*_CIT_, *bla*_ACC_, but positive for *bla*_TEM_, *bla*_OXA_, *bla*_CTX-M_, *intI1*, and *intI2* gene regions. Sequencing results of the *bla*_TEM_ showed 100% identity with *bla*_TEM-1_. All isolates harbored *bla*_OXA-1_, except the only one isolate from Shanghai. Thirteen isolates harbored *bla*_CTX-M-14_; the only one strain from Shanghai contained *bla*_CTX-M-79_. Notably, one isolate, collected from Xinjiang, simultaneously harbored both *bla*_CTX-M-28_ and *bla*_CTX-M-14_. All isolates, except for the Shanghai isolate, contained class 1 integrons, among which, three strains from Xinjiang and two strains from Yunnan harbored both the *bla*_OXA-30_ and *aadA1* gene cassettes. The *bla*_OXA-30_ and *bla*_OXA-1_ genes, which are contained on the Tn*1409* and Tn*2603* transposons respectively, differed by only a single nucleotide [17]. All 15 isolates harbored class 2 integrons with *dfrA1*, *sat1*, and *aadA1* gene cassettes.

**Table 6:** Antibiogram and molecular analysis of the antibiotic-resistance determinants and integrons of 15 cephalosporin resistant isolates.

Strain no.	Antibiogram	Antibiotic-resistant determinants and integrons present
SH13sh024	CAZ/CFP/CRO/CFZ/PIP/AMP/TIC/ATM	*IntI2* (*dfrA1+sat1+aadA1*), *bla*_TEM-1_, *bla*ctx-_M_-_79_
2010294	CFP/CRO/CFZ/PIP/AMP/TIC/TE/C/TIM	*IntI1* (*bla*_OXA-30_ *+aadA1*), *IntI2* (*dfrA1+sat1+aadA1*), *bla*_OXA-1_, *bla*_TEM-1_, *bla*_CTX_-_M_-_14_
2010297	CFP/CRO/CFZ/PIP/AMP/TIC/TE/C/TIM	*IntI1* (*bla*_OXA_-_30_ *+aadA1*), *IntI2* (*dfrA1+sat1+aadA1*), *bla*_OXA-1_, *bla*_TEM-1_, *bla*_CTX_-_M_-_14_ and _28_
2010315	CFP/CRO/CFZ/PIP/AMP/TIC/TE/C/TIM	*IntI1* (*bla*_OXA_-_30_ *+aadA1*), *IntI2* (*dfrA1+sat1+aadA1*), *bla*_OXA-1_, *bla*_TEM-1_, *bla*_CTX_-_M_-_14_
CDSF2	CFP/CRO/CFZ/PIP/AMP/TIC/TE/C/TIM/SXT	*IntI1*, *IntI2* (*dfrA1+sat1+aadA1*), *bla*_OXA-1_, *bla*_TEM-1_, *bla*_CTX_-_M_-_14_
CDSF3	CFP/CRO/CFZ/PIP/AMP/TIC/TE/C/TIM/SXT	*IntI1*, *IntI2* (*dfrA1+sat1+aadA1*), *bla*_OXA-1_, *bla*_TEM-1_, *bla*_CTX_-_M_-_14_
CDSF4	CFP/CRO/CFZ/PIP/AMP/TIC/TE/C/TIM/SXT	*IntI1* (*bla*_OXA-30_ *+aadA1*), *IntI2* (*dfrA1+sat1+aadA1*), *bla*_OXA-1_, *bla*_TEM-1_, *bla*_CTX_-_M_-_14_
CDSF5	CFP/CRO/CFZ/PIP/AMP/TIC/TE/C/TIM/SXT	*IntI1*, *IntI2* (*dfrA1+sat1+aadA1*), *bla*_TEM-1_, *bla*_CTX_-_M_-_14_
CDSF6	CFP/CRO/CFZ/PIP/AMP/TIC/TE/C/TIM/SXT	*IntI1*, *IntI2* (*dfrA1+sat1+aadA1*), *bla*_TEM-1_, *bla*_CTX_-_M_-_14_
CDSF7	CFP/CRO/CFZ/PIP/AMP/TIC/TE/C/TIM/SXT	*IntI1*, *IntI2* (*dfrA1+sat1+aadA1*), *bla*_OXA-1_, *bla*_TEM-1_, *bla*_CTX_-_M_-_14_
CDSF8	CFP/CRO/CFZ/PIP/AMP/TIC/TE/C/TIM/SXT	*IntI1*, *IntI2* (*dfrA1+sat1+aadA1*), *bla*_OXA-1_, *bla*_TEM-1_, *bla*_CTX_-_M_-_14_
CDSF9	CFP/CRO/CFZ/PIP/AMP/TIC/TE/C/TIM/SXT	*IntI1*, *IntI2* (*dfrA1+sat1+aadA1*), *bla*_OXA-1_, *bla*_TEM-1_, *bla*_CTX_-_M_-_14_
CDSF10	CFP/CRO/CFZ/PIP/AMP/TIC/TE/C/TIM/SXT	*IntI1* (*bla*_OXA-30_ *+aadA1*), *IntI2* (*dfrA1+sat1+aadA1*), *bla*_OXA-1_, *bla*_TEM-1_, *bla*_CTX_-_M_-_14_
CDSF11	CFP/CRO/CFZ/PIP/AMP/TIC/TE/C/TIM/SXT	*IntI1*, *IntI2* (*dfrA1+sat1+aadA1*), *bla*_OXA-1_, *bla*_TEM-1_, *bla*_CTX_-_M_-_14_
CDSF12	CFP/CRO/CFZ/PIP/AMP/TIC/TE/C/TIM/SXT	*IntI1*, *IntI2* (*dfrA1+sat1+aadA1*), *bla*_OXA-1_, *bla*_TEM-1_, *bla*_CTX_-_M_-_14_

### PFGE analysis

**Fig 1:**
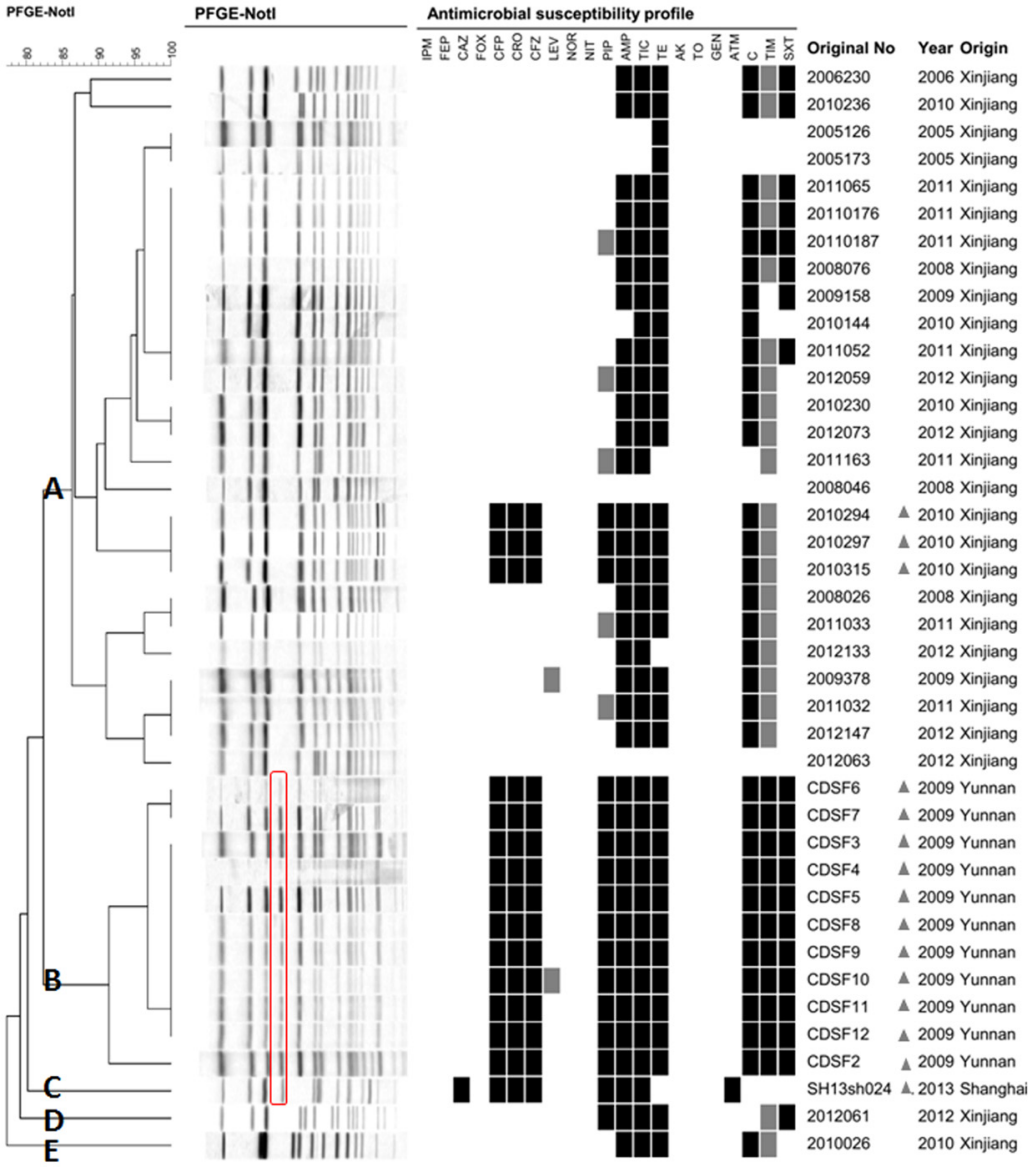
PFGE dendrogram and antibiotic resistance profile of 40 *S*. *flexneri* 1b isolates.

PFGE was performed to determine the genetic diversity of the *S*. *flexneri* 1b isolates. The 40 *S*. *flexneri* 1b isolates generated 18 PFGE patterns (Fig 1). All isolates could be divided into five main groups (A–E), with approximately 82% similarity. Notably, there was a high level of variation in the PFGE profiles of serotype 1b isolates between different regions of China, which contrasted with a previous study showing that 70 *S*. *flexneri* 1b strains isolated in Bangladesh shared the same PFGE profile [7]. In the present study, isolates from Xinjiang belonged to groups A, D, and E, whereas isolates from Yunnan and Shanghai belonged to groups B and C, respectively. Twenty-eight isolates from Xinjiang showed 14 different PFGE patterns; 26 of the 28 isolates formed a single cluster, with the two remaining isolates forming separate branches. Eleven isolates from Yunnan showed three different PFGE patterns, and the single isolate from Shanghai displayed a distinct PFGE pattern. Interestingly, the PFGE patterns of Xinjiang isolates were distinct from Yunnan and Shanghai isolates by one obvious, large, missing band indicated with a red border in Fig 1.

The original number, origin, and isolation year are indicated for each strain. The missing band of Xinjiang isolates was indicated with a red border and 15 cephalosporin resistant isolates were marked with triangles.

Among the isolates in different PFGE clusters, the prevalence of antibiotic resistance in clusters A, D, and E (Xinjiang) was much lower than that of isolates in cluster B (Yunnan). All 15 cephalosporin resistant isolates (marked with triangles in Fig 1) belonged to clusters B (Yunnan), C (Shanghai), and a small branch of cluster A (Xinjiang). All isolates in cluster B were resistant to cephalosporins, such as CFP, CRO, and CFZ, while the resistance rate of isolates in clusters A, D, and E (Xinjiang) to these antibiotics was 10.7%. Higher resistance rates to SXT, PIP, AMP, TIC, TE, C, and TIM were also observed in isolates from Xinjiang than in those from Yunnan. The isolate in cluster C (Shanghai) was the only isolate showing resistance to CAZ and ATM. In summary, similarities in resistance patterns were observed in strains with the same PFGE pattern.

## Discussion

Approximately 85% of the strains in our study showed MDR profiles, and were highly resistant to the older-generation antimicrobials, such as AMP, C, TE, and TIC. So the treatment should be based on susceptibility patterns and antimicrobials with current resistance may turn to be effective in the future. This rapid accumulation of resistance in *Shigella* is probably the result of inappropriate prescription of, and easy access to, antimicrobials among outpatients in China [18]. Besides, the PFGE dendrogram showed that the *S*. *flexneri* type 1b isolates from Yunnan are closely related (95% similarity), indicating that they are possibly derived from a common parental strain. In contrast, the isolates from Xinjiang shared lower degrees of similarity, suggesting that they are likely derived from divergent sources. The antibiotic susceptibility profiles of the isolates also varied between the geographical locations; the antimicrobial resistance situation in Xinjiang does not seem to be as serious as that in Yunnan and Shanghai. Among the 40 isolates, only the isolate from Shanghai was resistant to CAZ and ATM, and isolates from Yunnan were more resistant to antibiotics CFP, CRO, CFZ, PIP, AMP, TIC, TE, C, TIM, and SXT than those from Xinjiang and Shanghai. The diverse antibiotic susceptibility may be attributed to the fact that mainland China covers a large territory and has many nationalities, with more than 1.3 billion people living in diverse areas, hence many varying factors such as customs, climates, economies, geographies may affect the antimicrobial susceptibility patterns.

In the present study, all 15 cephalosporin resistant isolates harbored *bla*_CTX-M_ genes, most of which were *bla*_CTX-M-14_, followed by *bla*_CTX-M-79_, and *bla*_CTX-M-28_. A previous study indicated that plasmids carrying different *bla*_CTX-M_ genes were introduced into isolates at different times [18]. The *bla*_CTX-M-14_ subtype was identified in 14 of these isolates, which indicated that these genes might have been circulating among *Shigella* isolates for a long time. In addition, *bla*_TEM_ was detected in all 15 cephalosporin resistant isolates, and *bla*_OXA_ was amplified in all such isolates, except the isolate from Shanghai. OXA-type β-lactamases are characterized by high hydrolytic activity against cloxacillin and oxacillin, which confer resistance to cephalothin and ampicillin [19]. Notably, all *bla*_OXA_ isolates in this study carried *bla*_OXA-1_, suggesting a host specificity for this subtype in *S*. *flexneri*, which is consistent with a previous study by Siu et al. [17]. *bla*_OXA-30_ and *aadA1* were also detected in the gene cassettes of class 1 integrons in five of the 15 cephalosporin resistant isolates in our study. These two gene cassettes appear to be coordinately excised or integrated, as described by a previous study [3]. Pan et al. reported that class 2 integrons were present in 87.9% of *S*. *flexneri* isolates [14]. In our study, all of the 15 strains examined harbored class 2 integrons, which followed the *dfrA1+sat1+aadA1* gene cassette, conferring resistance to trimethoprim and streptomycin [20]. All of these various resistance genes facilitate the dissemination of resistance determinants and the survival of bacteria under the selective pressure of various antibiotics.

Fluoroquinolones and third-generation cephalosporins were two popular empirical options to treat severe gastrointestinal infections caused by pathogenic bacteria [21]. Ciprofloxacin is currently the first-line antibiotic recommended by the World Health Organization for treating shigellosis [22]. Fortunately, limited resistance to fluoroquinolones was observed in our study. The control of resistance to quinolones could be attributed to a relevant study on quinolone-induced cartilage toxicity [23]. Third-generation cephalosporins are considered alternative drugs for shigellosis treatment [22], but in our study, the isolates from Yunnan and Shanghai were all resistant to CFP, CRO, and CFZ, and the rate of resistance of isolates from Xinjiang to these cephalosporins was 10.7%. According to the literature, strains may acquire resistance to multiple antibiotics if exposed to a resistant gastrointestinal pathogen following antibiotic therapy [24]. Therefore, these ESBL-producing isolates, especially those from Yunnan and Shanghai, would put patients at high risk if third-generation cephalosporins were still used for empirical therapy. Moreover, the isolate from Shanghai also exhibited resistance to ATM and CAZ, which clearly raises the perennial issue of strict control of antimicrobial prescription in China.

In the present study, diverse antimicrobial resistance patterns were observed in different endemic areas, which support the need for prudent selection and use of antibiotics. Therefore, we suggest that antibiotic susceptibility testing should be performed at the start of an outbreak, and antibiotic use should be restricted to severe *Shigella* cases, based on variations in resistance patterns in different regions. Furthermore, resistance patterns should be recorded timely and locally, and empiric antibiotic therapy for *Shigella* cases should be changed accordingly. The data that we obtained here might provide a strategy for the treatment of infections caused by *S*. *flexneri* serotype 1b strains in China.
